# Evaluation of hypotension prediction index software in patients undergoing orthotopic liver transplantation: retrospective observational study

**DOI:** 10.1016/j.bjane.2025.844589

**Published:** 2025-01-22

**Authors:** Jacek B. Cywinski, Yufei Li, Lusine Israelyan, Roshni Sreedharan, Silvia Perez-Protto, Kamal Maheshwari

**Affiliations:** a Cleveland Clinic, Department of General Anesthesiology, Cleveland, Ohio; b Cleveland Clinic, Department of Outcome Research, Cleveland, Ohio; c Cleveland Clinic, Department of Quantitative Health Sciences, Cleveland, Ohio; d Cleveland Clinic, Department of Intensive Care and Resuscitation, Cleveland, Ohio

**Keywords:** Cardiac output, Hypotension prediction index, Intraoperative hypotension, Liver transplantation, Systemic vascular resistance

## Abstract

**Background:**

Extreme hemodynamic changes, especially intraoperative hypotension (IOH), are common and often prolonged during Liver Transplant (LT) surgery and during initial hours of recovery. Hypotension Prediction Index (HPI) software is one of the tools which can help in proactive hemodynamic management. The accuracy of the advanced hemodynamic parameters such as Cardiac Output (CO) and Systemic Vascular Resistance (SVR) obtained from HPI software and prediction performance of the HPI in LT surgery remains unknown.

**Methods:**

This was a retrospective observational study conducted in a tertiary academic center with a large liver transplant program. We enrolled 23 adult LT patients who received both Pulmonary Artery Catheter (PAC) and HPI software monitoring. Primarily, we evaluated agreement between PAC and HPI software measured CO and SVR. A priori, we defined a relative difference of less than 20% between measurements as an adequate agreement for a pair of measurements and estimated the Lin's Concordance Correlation Coefficient and Bland-Altman Limits of Agreement (LOA). Clinically acceptable LOA was defined as ± 1 L.min^-1^ for CO and ± 200 dynes s.cm^-5^ for SVR. Secondary outcome was the ability of the HPI to predict future hypotension, defined as Mean Arterial Pressure (MAP) less than 65 mmHg lasting at least one minute. We estimated sensitivity, positive predictive value, and time from alert to hypotensive events for HPI software.

**Results:**

Overall, 125 pairs of CO and 122 pairs of SVR records were obtained from 23 patients. Based on our predefined criteria, only 42% (95% CI 30%, 55%) of CO records and 53% (95% CI 28%, 72%) of SVR records from HPI software were considered to agree with those from PAC. Across all patients, there were a total of 1860 HPI alerts (HPI ≥ 85) and 642 hypotensive events (MAP < 65 mmHg). Out of the 642 hypotensive events, 618 events were predicted by HPI alert with sensitivity of 0.96 (95% CI: 0.95). Many times, the HPI value remained above alert level and was followed by multiple hypotensive events. Thus, to evaluate PPV and time to hypotension metric, we considered only the first HPI alert followed by a hypotensive event (“true alerts”). The “true alert” was the first alert when there were several alerts before a hypotension. There were 614 “true alerts” and the PPV for HPI was 0.33 (95% CI 0.31, 0.35). The median time from HPI alert to hypotension was 3.3 [Q1, Q3: 1, 9.3] mins.

**Conclusion:**

There was poor agreement between the pulmonary artery catheter and HPI software calculated advanced hemodynamic parameters (CO and SVR), in the patients undergoing LT surgery. HPI software had high sensitivity but poor specificity for hypotension prediction, resulting in a high burden of false alarms.

## Background

Extreme hemodynamic changes are common and often prolonged during Liver Transplant (LT) surgery and during initial hours of recovery. Advanced hemodynamic monitoring including Pulmonary Artery Catheter (PAC) and Transesophageal Echocardiography (TEE) are often used intraoperatively to optimize blood pressure, volume status and cardiac output. Nonetheless, hyperdynamic circulation, low Systemic Vascular Resistance (SVR), significant blood loss, coagulopathy and surgical manipulation of vascular structures pose a significant challenge for the anesthesia care team to maintain stable hemodynamic status. The risk of hemodynamic instability remains in the immediate postoperative period and during the intensive care unit stay. Therefore, it is important to monitor patients closely, anticipate hemodynamic changes and intervene with fluid, vasopressors or inotropic agents, in a timely manner to mitigate cardiovascular disturbances.

Most hemodynamic perturbations are manifested as systemic arterial hypotension, which can result from inadequate intravascular volume, vasodilation, myocardial dysfunction, or a combination of all three elements. Recent evidence suggests that even short periods of Intraoperative Hypotension (IOH) are associated with worse postoperative outcomes. For example, IOH defined as mean cumulative arterial blood pressure (MAP) < 65 mmHg lasting for more than 15 mins or MAP < 55 mmHg for a few minutes was associated with increased mortality, incidence of Acute Kidney Injury (AKI) and myocardial injury; similar findings were confirmed in ICU settings.^1^, ^2^, ^3^ However, current hemodynamic management is mostly reactive, and the hypotension is treated once it has already occurred.

Hypotension Prediction Index (HPI) software provides a capability of hypotension episode prediction in the future 5‒15 minutes empowering clinicians to proactively manage hemodynamics.^4^ The HPI software utilizes a sophisticated analysis of arterial line waveform to predict future hypotensive events.^5^ Arterial waveform time, amplitude, area, segment slopes, and complexity are used to predict hypotension, defined as MAP less than 65 mmHg lasting for at least 1 min. The Index Values (HPI) range from 0 to 100, with higher numbers reflecting a higher likelihood of subsequent hypotension. The index reportedly has 92% sensitivity and specificity for predicting hypotension 5 min in advance; sensitivity 89% and specificity 90% for 10 min, and 88% and 87% for 15 min prediction in advance, respectively.^6^ The software is now FDA approved and is the only commercially available platform in the United States to predict hypotension. The advanced hemodynamic parameters like cardiac output, stroke volume, and dynamic elastance provided in addition to HPI can inform about the root cause of hypotension and guide appropriate intervention.

This technology was developed and validated in clinical trials, and it seems to be reasonably accurate in the general surgical population undergoing major surgical procedures.^6^^,^^7^ However, the utility of HPI in hemodynamic management of LT patients is unknown. Also, it is unclear if implementation of HPI software into clinical practice reduces the incidence of IOH; studies published thus far demonstrated mixed results.^5^^,^^8^

In this retrospective study, we evaluated the correlation of advanced hemodynamic variables between PAC and HPI software (specifically CO and SVR) as well as the performance of HPI prediction during and after surgery in patients undergoing liver transplantation.

Primarily, we evaluated the agreement between PAC derived CO and SVR and the same calculated by HPI software during and after LT surgery.

Secondarily, we evaluated the ability of HPI software to predict hypotensive episodes defined as MAP < 65 mmHg lasting for at least 1 minute during LT surgery and the first 24 hours of recovery in intensive care unit. We evaluated the number of alerts, time to hypotension from HPI alert, and overall hypotension burden during the observation period.

## Methods

This was a retrospective review to verify the agreement between PAC and HPI software (HemoSphere with Acumen IQ sensor platform ‒ Edwards Lifesciences Corp. One Edwards Way Irvine, CA 92614) on measurements of the same parameters (CO and SVR) at the same time points.^4^ Performance of the HPI software was also evaluated for its prediction of intraoperative and postoperative arterial hypotension. The study was approved by Institutional Review Board (IRB# 21-287), which waived the need for informed consent. HemoSphere with Acumen IQ sensor monitor was introduced to clinical practice at our institution on November 1, 2020, as an addition to other hemodynamic monitors (arterial line, PAC, and TEE) during LT surgery and during the first 24-hour stay in Surgical Intensive Care Unit (SICU) after surgery.

Medical records from 24 patients, who underwent LT between November 1^st^, 2020, and February 28^th^, 2021, and were monitored intraoperatively by both PAC and HPI software, were reviewed individually. Hemodynamic data from the HPI software was offloaded from the monitor to an encrypted jump drive after each case and stored on a dedicated password protected computer for the analysis. Hemodynamic data from PAC were obtained from Anesthesia Record Keeping software (ARKS), stored in Perioperative Health Data System (PHDS database) and Surgical Intensive Care Patient Database Registry, both IRB approved. Agreement between PAC and HPI software was evaluated using only intraoperative data due to the lack of PAC data in the SICU. Performance of HPI software in predicting future episodes of hypotension was assessed using data from the whole HPI monitoring period, which covered the LT surgery and the first 24 hours of stay in the SICU.

### Data analysis

Patients’ baseline characteristics, including age, gender, BMI, Chemical Model for End-stage Liver Disease (MELD) score, surgery time, intraoperative blood loss, intraoperative transfusion requirement, intraoperative fluids, intraoperative vasoactive medications, and intraoperative urine output were summarized using standard descriptive statistics.

Agreement between HPI software and PAC was assessed based on two outcomes: Cardiac Output (CO) and Systemic Vascular Resistance (SVR). We considered PAC measurement as a reference, and predefined ± 1 L.min^-1^ as the Clinically Acceptable Difference (CAD) for CO and ± 200 dynes sec.cm^-5^ as the CAD for SVR. We arbitrarily defined ±1 L.min^-1^ as the Clinically Acceptable Difference (CAD) for CO based on pragmatic, clinically relevant accuracy necessary for decision making in patients undergoing liver transplant. Measurements from PAC and HPI software of the same outcome were compared for each patient at the same time point during surgery.

The Concordance Correlation Coefficient (CCC), which was used to summarize the agreement between two measurements by measuring the departure of their linear relationship from the 45-degree diagonal line, was estimated first and bootstrap resampling with replacement was used to estimate its confidence interval, adjusting for within-patient correlation. Specifically, Lin's CCC was estimated using the original data.^9^ Then, the original data was resampled at the patient level with replacement (23 patients were randomly selected from a total of 23 patients with replacement, since one patient had missing intraoperative data). The resampled patients were then merged with each patient's data for analysis. The 2.5^th^ and 97.5^th^ percentiles of the distribution of the CCCs from the 1,000 resamples were used to estimate the 95% Confidence Interval (95% CI) of CCC.

For each outcome, the average of repeated differences between PAC and HPI software was calculated for each patient. The mean of individual average differences (estimate bias) and the SD of bias were summarized across patients using the Bland-Altman's Limit of Agreement (LOA) method for repeated measures, adjusting for within-patient correlation.^10^ The 95% LOA was computed as bias ± 1.96* D. The 95% Confidence Intervals for LOAs were estimated using the Method of Variance Estimates Recovery (MOVER).^11^ Individual paired measurement differences were plotted against the average of the two measurements to assess any trend towards changing variability of the difference with changing mean.

In practice, we would consider HPI software and PAC to agree with each other if they had a relative difference of measuring the same outcome at the same time less than 20%. The relative difference was calculated as the absolute value of (PAC – HPI software) / PAC × 100. We estimated the proportion of relative difference less than 20% by fitting data with an intercept-only GEE model to adjust for within-patient correlation for repeated measures. The proportion of relative differences less than 20% and its 95% CI were calculated from the estimated model intercept and SE.

### Sample size considerations

We included all available patients in our analysis. A post hoc power analysis was conducted based on the final number of measurement pairs. For cardiac output measurements (n = 125 pairs), using a predefined Clinically Acceptable Difference (CAD) of ± 1 L.min^-1^ and assuming a mean difference of zero, we calculated that the study would have at least 87% power to conclude agreement if the standard deviation of the differences was not more than 0.4, based on the Bland-Altman limits of agreement method.

Similarly, for systemic vascular resistance measurements (n = 122 pairs), with a predefined CAD of ± 200 dynes·sec.cm^-5^ and assuming a mean difference of zero, the study would have at least 80% power to conclude agreement if the standard deviation of the differences was not more than 81.

For the secondary analysis, episodes of intraoperative and postoperative hypotension were defined as MAP < 65 mmHg for at least 1 minute continuously. A hypotension episode started when MAP < 65 mmHg and ended when MAP ≥ 65 mmHg, with all MAP records within the episode lower than 65 mmHg. Episodes of HPI alert were defined as at least two continuous records of HPI ≥ 85. An alert episode started when HPI ≥ 85 mmHg and ended when HPI < 85, with all HPI records within the episode higher than or equal to 85.

**Figure 1:**
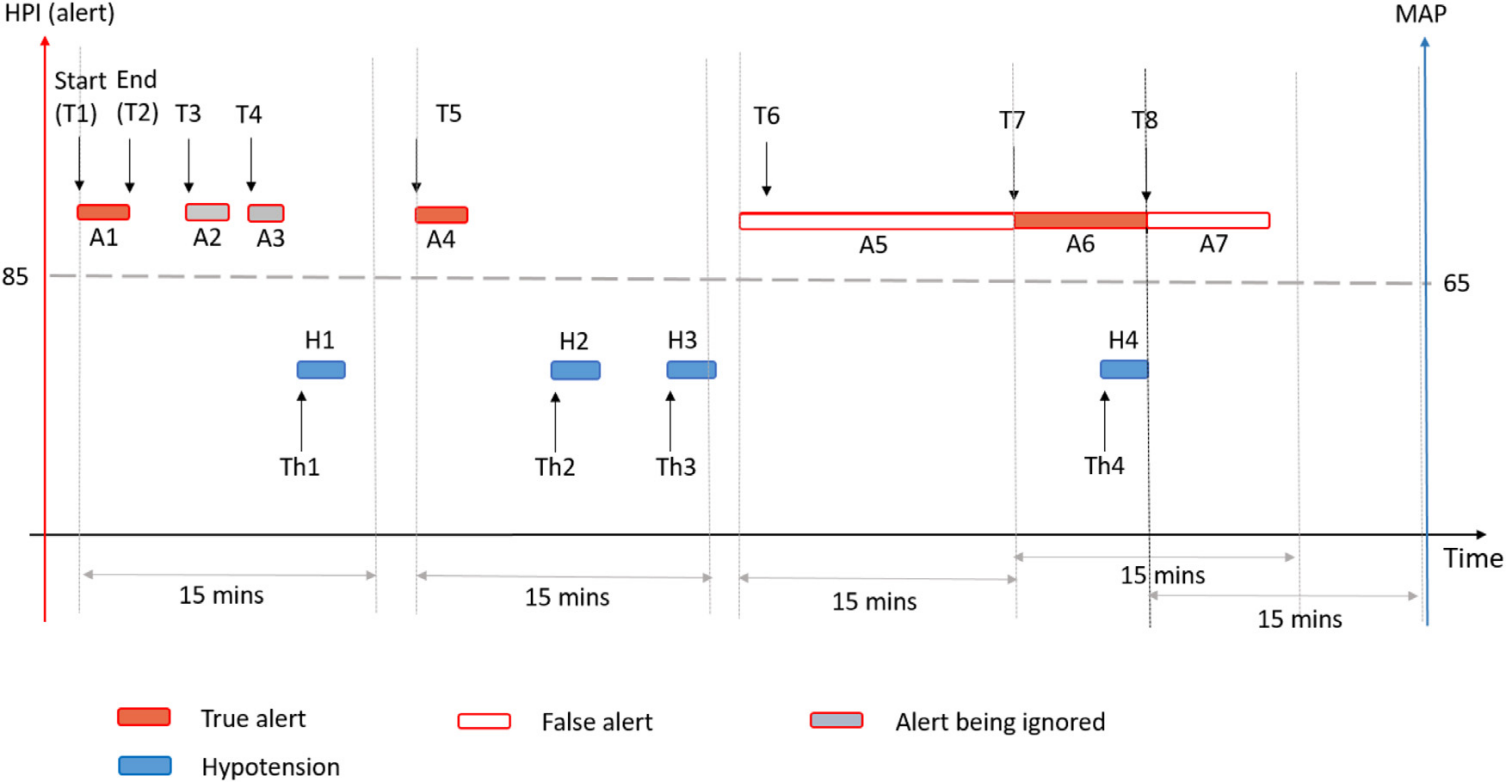
Prediction alert and hypotension episode. Hypotension episode: MAP < 65 mmHg for at least 1 minute continuously. Started when MAP < 65 mmHg and ended when MAP ≥ 65 mmHg. Alert episode: at least two continuous records of HPI ≥ 85. Started when HPI ≥ 85 and ended when HPI < 85. From the start of an alert episode, if there was hypotension within 15 mins, we considered this alert to have successfully predicted the subsequent hypotension and called it a “true alert”. A “false alert”, by contrast, was defined when no hypotension occurred within 15 mins. If there were multiple subsequent alert episodes (A1, A2, and A3) before the first hypotension (H1), the subsequent alert episodes (A2 and A3) were ignored. If there were multiple subsequent hypotension episodes (H2 and H3) within 15 mins (from T5, the start of A4), all hypotension episodes (H2 and H3) were predicted by this single HPI alert (A4). For alerts lasting more than 15 mins or when hypotension occurred within the alert episode, we split the alert episode either at 15 mins (T7) when no hypotension occurred; or at the end of the subsequent hypotension episode (T8) when a Hypotension episode (H4) occurred within 15 mins. Time to hypotension was defined as the duration from the start of the true alert episode to the start of the predicted hypotension episode (Th1–T1). For alerts that predicted multiple hypotension episodes, time to the first hypotension was counted (Th2–T5).

From the start of an alert episode, if there was a hypotension episode within 15 mins, we considered this alert to have successfully predicted the subsequent hypotension and called it a “true alert”. A “false alert”, by contrast, was defined when no hypotension occurred within 15 mins of the HPI alert. If there were multiple subsequent alert episodes before the first hypotension, the subsequent alert episode was ignored. If there were multiple subsequent hypotension episodes within 15 mins, all hypotension episodes were predicted by this single HPI alert. For alerts lasting more than 15 mins or when hypotension occurred within the alert episode, we split the alert episode either at 15 mins (when no hypotension occurred) or at the end of the first subsequent hypotension episode (when there was hypotension within 15 mins) (Fig. 1).

We estimated the sensitivity of the HPI software as the number of predicted hypotension episodes divided by the total number of hypotension episodes. The 95% CI for sensitivity was estimated using$\;sensitivity\pm Z_{1-\alpha /2}\sqrt{\frac{sensitivity\;\left(1-sensitivity\right)}{n}}$, where n is the total number of hypotension episodes.

The Positive Predictive Value (PPV) of the HPI software was estimated using the number of true alerts divided by the total number of HPI alerts. The 95% CI for PPV was estimated using$\;ppv\pm Z_{1-\alpha /2}\sqrt{\frac{ppv(1-ppv)}{n}}$, where n is the total number of HPI alert episodes.

We also summarized the time to hypotension episode from HPI alert (Fig. 1). Time to hypotension episode was only calculated for true alerts and was defined as the duration from the start of the true alert episode to the start of the predicted hypotension episode. For alerts that predicted multiple hypotension episodes, only time to the first hypotension episode was counted.

Hypotension severity was characterized as the Area Under the Curve of MAP less than 65 mmHg (AUC-MAP), minutes of MAP less than 65 mmHg, and Time-Weighted Average MAP (TWA-MAP). We evaluated the severity with three thresholds: 65 mmHg, 60 mmHg, and 55 mmHg. AUC-MAP (mmHg.min^-1^) below each threshold was calculated as the cumulative sum of the areas below the given threshold for a patient using the trapezoid rule. MAP measurements were recorded every 20s by the HemoSphere system from the arterial catheter. Calculation of a specific area started when MAP was less than 65 mmHg and ended when MAP was greater than 65 mmHg. TWA-MAP representing the average (over time) mmHg below the threshold was calculated by dividing AUC-MAP by the total measurement time.

## Results

We enrolled 23 patients monitored by both PAC and HPI software during OLT surgery and SICU stay in this study. Patients’ demographic and perioperative characteristics were summarized in Table 1.

**Table 1:** Patient characteristics.

Factor	Total (n = 23)
Age	57.5 ± 10.4
Gender (Female)	9 (37.5)
BMI	28.8 ± 6.8
MELD Score	19.4 ± 8.2
Surgery Time (min)	684.2 ± 162.6
Intraoperative Vasoactive Medications	
Norepinephrine (mg)	4.4 [1.9, 5.9]
Vasopressin (mg)	8.0 [1.4, 17.0]
Epinephrine (mg)	0.02 [0.00, 0.06]
Intraoperative Fluids	
Colloids (cc)	5000.0 [2875.0, 5500.0]
Crystalloids (cc)	2000.0 [1725.0, 3000.0]
Total (cc)	6750.0 [5000.0, 8175.0]
Intraoperative Blood Loss (cc)	3000.0 [1400.0, 5000.0]
Intraoperative Transfusion	
Cryoprecipitate (cc)	350.0 [0.00, 1000.0]
FFP (cc)	600.0 [0.00, 1200.0]
Platelets (cc)	500.0 [250.0, 750.0]
RBC (cc)	1925.0 [1400.0, 2800.0]
Total (cc)	3975.0 [2125.0, 4750.0]
Urine (cc)	950.0 [681.5, 1297.5]

SD-Standard Deviation; Statistics presented as Mean ± SD, Median [p25, p75] or N (column %).

MELD score-Model for End-stage Liver Disease, MELD score is calculated based on serum bilirubin, serum creatinine, and the international normalized ratio for prothrombin time; FP, Frozen Plasma; RBC, Red Blood Cell.

**Figure 2:**
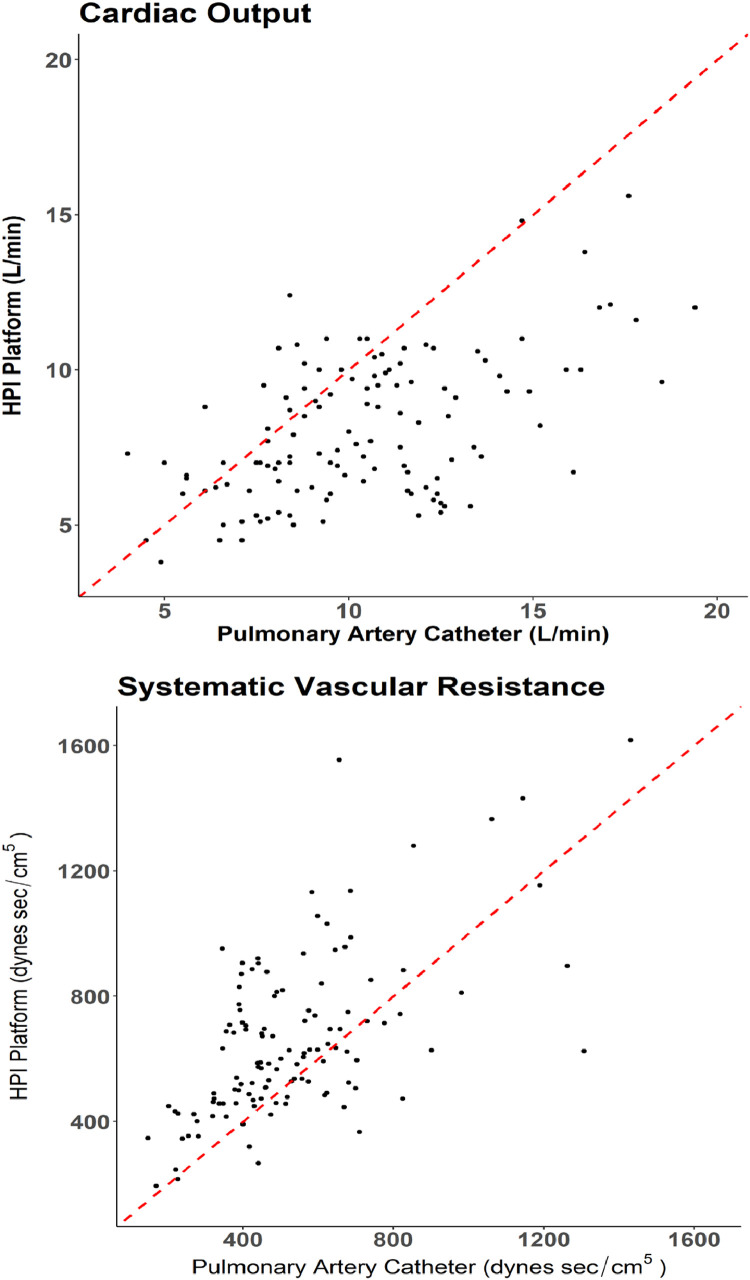
Scatter plots of HPI software against the reference pulmonary artery catheter measurement on cardiac output and systemic vascular resistance. The Red dashed line is the 45-degree diagonal line, which indicates the perfect concordance of the two measurements. Plots were based on intraoperative measurements from 23 patients, with 125 pairs of cardiac output records and 122 pairs of systemic vascular resistance records.

Agreement analyses between HPI software and PAC were based on data from 23 patients (one patient had missing intraoperative data), with 2‒11 replicates per individual. A total of 125 pairs of intraoperative CO records and 122 pairs of intraoperative SVR records from HPI software and PAC were compared (Table 2). The overall CO measurements from HPI software were lower compared to PAC, with the majority of points below the 45-degree diagonal line, while the overall SVR measurements from HPI software were higher compared to PAC, with the majority of points above the 45-degree line (Fig. 2). The estimated CCC for CO and SVR were 0.37 (95% CI: 0.13, 0.56) and 0.53 (95% CI: 0.28, 0.72), respectively (Table 3). Thus, there was poor agreement between PAC and HPI software on measuring CO and SVR.

**Table 2:** Summary of cardiac output and systemic vascular resistance.

Outcome	Mean ± SD	Min	Q1	Median	Q3	Max	n
Cardiac Output (L.min^-1^) (n = 125)^a^							
PAC	10.4 ± 3.1	4.0	8.1	10.2	12.3	19.4	125
HPI software	8.1 ± 2.3	3.8	6.2	7.5	9.7	15.6	125
Difference (PAC – HPI software)	2.3 ± 2.7	−4.0	0.4	2.0	4.0	9.4	125
Systemic Vascular Resistance (dynes sec.cm^-5^) (n = 122)^a^							
PAC	531 ± 228	147	393	477	626	1431	122
HPI software	657 ± 260	194	474	612	770	1618	122
Difference (PAC – HPI software)	−126 ± 218	−898	−285	−110.5	−4	682	122

PAC, Pulmonary Artery Catheter.

Intraoperative cardiac output and systemic vascular resistance measurements from 23 patients, with 2‒11 replicates per individual. Summary statistics were generated ignoring the within-patient correlation.

a Number of matched pairs.

**Table 3:** Agreement between Pulmonary Artery Catheter (PAC) and HPI software.^a^

Measure	Cardiac Output	Systematic Vascular Resistance
Bias (mean ± SD)^b^	1.96 ± 2.74	−93 ± 241
95% Limits of agreement^c^		
Upper (95% CI)	7.34 (6.26, 8.92)	379 (270, 543)
Lower (95% CI)	−3.42 (−5.00, −2.34)	−565 (−729, −456)
Proportion of relative differences < 20% (95% CI)^d^	0.42 (0.30, 0.55)	0.37 (0.27, 0.49)
CCC (95% CI)^e^	0.37 (0.13, 0.56)	0.53 (0.28, 0.72)

a 125 Pairs of cardiac output records and 122 pairs of systemic vascular resistance records from 23 patients.

b Bias was calculated as the average difference between PAC and HPI software across all measurements for each patient. Bland-Altman limits of agreement were calculated using bias ± 1.96 Standard Deviation (SD), adjusting for within-patient correlation.

c Bland-Altman limits of agreement for repeated measures, adjusting for within-patient correlation. The 95% Confidence Intervals (95% CIs) for limits were estimated using the method of variance estimates recovery (MOVER) [ZY Zou, 2011].

d Proportion and 95% CI were estimated by fitting the intercept-only GEE model, adjusting for within-patient correlation.

e 95% CI for Concordance Correlation Coefficient (CCC) was estimated using the bootstrap percentile method based on 1,000 bootstrapping resamples with replacement.

**Figure 3:**
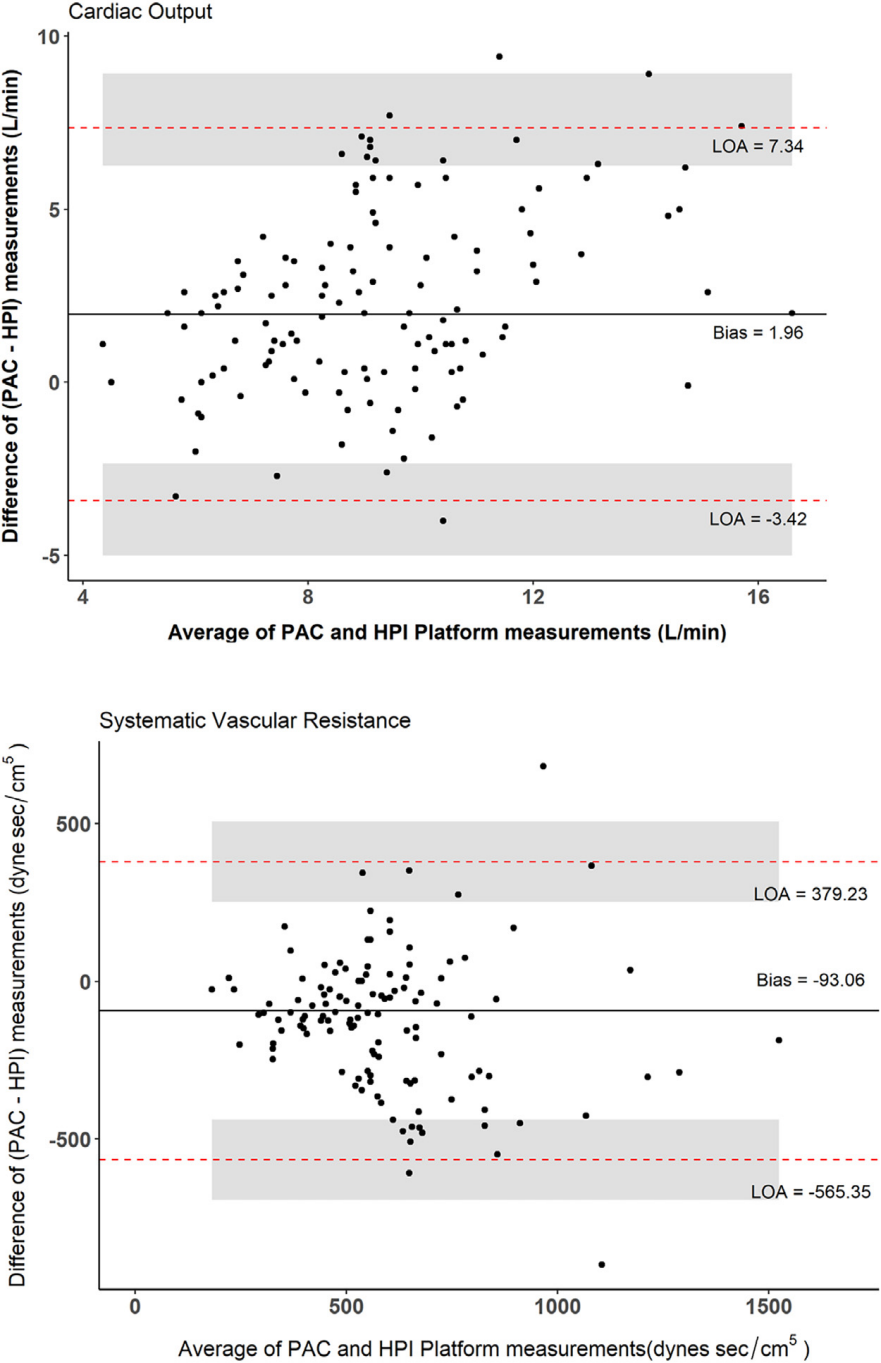
Bland-Altman plots for HPI software vs. the reference Pulmonary Artery Catheter (PAC) measurement on cardiac output and systemic vascular resistance. The X-axis is the average of the two measurements, the Y-axis is the difference between the two measurements, specifically, PAC – HPI software. The solid line is the mean bias; red dash lines are 95% Limits of Agreement (LOAs); shaded areas represent the 95% Confidence Interval (95% CI) for limits of agreement. Plots were based on intraoperative measurements from 23 patients, with 125 pairs of cardiac output records and 122 pairs of systemic vascular resistance records. Bias and LOAs were calculated using Bland-Altman limits of agreement for repeated measures, adjusting for within-patient correlation. The 95% CIs for limits were estimated using the Method of Variance Estimates Recovery (MOVER).^8^

Bland-Altman limits of agreement analysis showed a bias of 1.96 L.min^-1^ (SD = 2.74 L.min^-1^) for CO. The 95% limits of agreement were -3.42 L.min^-1^ (95% CI: -5.00, -2.34) and 7.34 L.min^-1^ (95% CI: 6.26, 8.92), which were outside of our *a priori* defined CAD range of -1 to 1 L.min^-1^. Thus, the HPI software did not agree with PAC sufficiently. The bias for SVR was -93 dynes sec.cm^-5^ (SD = 241 dynes sec.cm^-5^), with 95% LOA of -565 dynes sec.cm^-5^ (95% CI: -729, -456) and 379 dynes sec.cm^-5^ (95% CI: 270, 543). This was also outside of the *a priori* defined CAD range ( [-200, 200] dynes sec.cm^-5^) for SVR (Fig. 3 and Table 3).

We considered HPI software to agree with PAC if the relative difference of measuring the same outcome at the same time was less than 20%, using PAC measurement as a reference. The proportion of CO records with the relative difference between PAC and HPI software less than 20% was 0.42 (95% CI: 0.30, 0.55), while that for SVR was 0.37 (95% CI: 0.27, 0.49) (Table 3).

The median duration of MAP less than 65 mmHg was 117 [Q1, Q3: 44, 295] min, the median AUC was 301 [Q1, Q3: 168, 1177] mmHg.min^-1^, and the median time-weighted average MAP less than 65 mmHg was 0.43 [Q1, Q3: 0.17, 1.03] mmHg.

Across all patients, there were a total of 1860 HPI alerts (HPI ≥ 85) and 642 hypotensive events (MAP < 65 mmHg). Out of the 642 hypotensive events, 618 events were predicted by HPI alert. The sensitivity of HPI alert was 0.96 (95% CI: 0.95, 0.98). Some alerts predicted multiple hypotensive events (Fig. 1). Thus, the number of “true alerts”, which had successfully predicted subsequent hypotension episode(s), was less than the number of hypotension events being predicted. There were 614 “true alerts” and the PPV for HPI was 0.33 (95% CI: 0.31, 0.35). The median time from HPI alert to hypotension was 3.3 [Q1, Q3: 1, 9.3] mins.

Overall, HPI software showed a poor to weak agreement with PAC in terms of CCC and Bland Altman limits of agreement. Only 42% (95% CI: 30%, 55%) of CO records and 53% (95% CI: 28%, 72%) of SVR records from HPI software were considered to agree with those from PAC. HPI software had a sensitivity of 90% (95% CI: 88%, 93%), and a positive predictive value of 33% (95% CI: 30%, 36%) for prediction of hypotension.

## Discussion

Our study demonstrated that HPI software had poor agreement of CO and SVR with gold standard PAC during liver transplantation. HPI software had high sensitivity but poor specificity for hypotension prediction, resulting in high burden of false alarms.

There is growing body of evidence that IOH is associated with adverse postoperative outcomes in patients undergoing non cardiac surgery.^12^ Less is known about the effect of IOH in LT recipients, however one recently published retrospective analysis demonstrated that the exposure to IOH was indeed associated with the increased risk of postoperative AKI.^13^ Even though the causality has yet to be established between IOH and adverse outcomes, it makes sense that IOH should be avoided based on a very plausible physiologic mechanism of the injury, dose response curve and temporal correlation.^1^ The ability to accurately measure advanced hemodynamic parameters in real time may help to identify the cause of the hemodynamic instability (most commonly manifested as IOH) and guide appropriate treatment. However, many continuous hemodynamic monitors, relying on the analysis of the arterial waveform, failed to be accurate in the patients in end stage liver disease and undergoing LT.^14^ Perhaps this is because of the unique changes in the cardiovascular system seen in this patient population: hyperdynamic circulation, low SVR, high cardiac output, splanchnic vasodilatation just to mention a few. Also, it remains unknown if HPI software can reliably predict future episodes of systemic hypotension in these patients as it is based on the analysis of arterial wave form features.^6^ In the past, few studies evaluated minimally invasive cardiac output monitors deriving the hemodynamic measurements from arterial pulse contour; especially uncalibrated devices largely showed poor correlation and interchangeability with the PAC during LT. Thus, our findings are not surprising.^15^, ^16^, ^17^

On the other hand, it seems that the ability of the HPI software to predict future episodes of hypotension was quite strong, potentially giving the anesthesiologist time to intervene before the IOH occurs. Conceivably, utilization of HPI information and acting upon the alerts could potentially decrease patient exposure to IOH, but the validity of that assumption needs further determination. However, its significant limitation lies in the high number of false positive alerts. Excessive number of false alarms may lead to unnecessary follow-up actions, alarm fatigue or distraction from other critical tasks.

So far, data from the studies in non-LT patients (non-cardiac surgery) demonstrated that HPI along with individualized goal directed therapy can reduce the incidence of IOH if a specific intervention protocol is followed by anesthesia providers, but others showed no benefit of HPI in reducing IOH burden.^5^^,^^8^^,^^18^^,^^19^

Interestingly Maheshwari et al. demonstrated in the sub-analysis of the Crystalloid versus Colloid Administration on Major Postoperative Morbidity trial, that the relationship between MAP and a composite of serious perfusion-related complications is largely independent of cardiac index in patients undergoing major abdominal surgery.^20^ Since most of the patients undergoing LT have either normal or supranormal CO, the maintenance of adequate perfusion pressure and avoidance of IOH may be more important than maintenance or augmentation of CO. In that respect, the value of the HPI software, when used during LT surgery, could be related to the prediction and proactive treatment of future hypotensive episodes rather than precise measurement of CO or SVR. It still needs to be determined in prospective trials if the results/data suggested by the HPI software hemodynamic interventions to prevent IOH are accurate in patients undergoing LT, because they are based on the measurements of CO and SVR, which had poor agreement with PAC measurements in our patients.

This retrospective study has important limitations related to the small number of included patients and lack of the management protocol to document if the response to HPI alerts indeed reduces the patient's exposure to IOH. Also, because of the retrospective nature of the study, we did not control the frequency of CO measurements with PAC, and it is possible, that under certain circumstances, agreement between the two measurement methods was better.

In our opinion, this investigation, within its limitations, showed that the HPI platform is inaccurate to measure CO and SVR, however it may have a role in reducing the exposure to IOH in patients undergoing LT. It is important to note that our analysis did not account for intraoperative interventions to treat hypotension, and clinicians were likely to use other clinical parameters predicting impeding hypotension. This could have contributed to the high number of false positives.

While the use of HPI demonstrates its benefit in detecting hypotension, its major limitation is the high number of false positives. Excessive false alerts may lead to unnecessary follow-up actions or treatments by clinicians, potentially reducing efficiency and diverting attention from other critical tasks. Whether the potential benefit of HPI software (early detection of hypotension) will translate into improved outcomes remains to be determined in prospective trials.

## Conclusion

There was poor agreement between the pulmonary artery catheter and HPI platform calculated advanced hemodynamic parameters, specifically CO and SVR, in patients undergoing LT. HPI software had high sensitivity but poor specificity for hypotension prediction, resulting in high burden of false alarms. While HPI demonstrates its benefit in detecting hypotension, its limitation lies in the high number of false positives. Excessive false positives may lead to unnecessary follow-up actions or treatments by clinicians, potentially reducing efficiency and diverting attention from other critical tasks. The role of HPI software in reducing IOH and improving outcomes deserves further studies.

## Authors’ contributions

Jacek B. Cywinski: This author helped to conduct the study, with data collection, data analysis, and manuscript preparation.

Yufei Li: This author helped with the data collection, data analysis, and manuscript preparation.

Lusine Israelyan: This author helped to conduct the study, with data collection and manuscript preparation.

Roshni Sreedharan: This author helped to conduct the study, with data collection, and manuscript preparation.

Silvia Perez-Proto: This author helped to conduct the study, with data collection, and manuscript preparation.

Kamal Maheshwari: This author helped to conduct the study, with data collection, data analysis, and manuscript preparation.

## Declaration of competing interest

The authors declare no conflicts of interest.
